# Regulation of Intertidal Microphytobenthos Photosynthesis Over a Diel Emersion Period Is Strongly Affected by Diatom Migration Patterns

**DOI:** 10.3389/fmicb.2016.00872

**Published:** 2016-06-07

**Authors:** Paulo Cartaxana, Sónia Cruz, Carla Gameiro, Michael Kühl

**Affiliations:** ^1^Marine Biological Section, Department of Biology, University of CopenhagenHelsingør, Denmark; ^2^Departamento de Biologia and Centro de Estudos do Ambiente e do Mar, Universidade de AveiroAveiro, Portugal; ^3^Centro de Ciências do Mar e Ambiente, Faculdade de Ciências, Universidade de LisboaLisboa, Portugal; ^4^Plant Functional Biology and Climate Change Cluster, University of Technology Sydney, UltimoNSW, Australia

**Keywords:** microsensors, diatoms, migration, chlorophyll fluorescence, photoacclimation

## Abstract

Changes in biomass and photosynthesis of a diatom-dominated microphytobenthos (MPB) intertidal community were studied over a diel emersion period using a combination of O_2_ and scalar irradiance microprofiling, variable chlorophyll (Chl) fluorescence, and pigment analysis. The MPB biomass in the photic zone (0–0.5 mm) of the sediment exposed to low irradiance (150 μmol photons m^-2^ s^-1^) showed *a* >2-fold increase during the first hours of the emersion period, reaching >0.2 mg Chl *a* cm^-3^. Concentrations of Chl *a* started to decrease half-way through the emersion period, almost 2 h before tidal inundation. Similarly, O_2_ concentrations and volumetric gross photosynthesis in the photic zone increased during the first half of the emersion period and then decreased toward the timing of incoming tide/darkness. The results suggest that intertidal MPB community-level photosynthesis is mainly controlled by changes in the productive biomass of the photic zone determined by cell migration. A diel pattern in the photosynthesis vs. irradiance parameters *α* (photosynthetic efficiency at limiting irradiance) and *ETR*_max_ (photosynthetic capacity at saturating irradiance) was also observed, suggesting photoacclimation of MPB. Under high light exposure (2000 μmol photons m^-2^ s^-1^), lower *α*, *ETR*_max_ and sediment O_2_ concentrations were observed when cell migration was inhibited with the diatom motility inhibitor latrunculin A (Lat A), showing that migration is also used by MPB to maximize photosynthesis by reducing exposure to potentially photoinhibitory light levels. A higher de-epoxidation state in sediment treated with Lat A indicates that the involvement of the xanthophyll cycle in physiological photoprotection is more relevant in MPB when cells are inhibited from migrating. In the studied diatom-dominated MPB intertidal community, cell migration seems to be the key factor regulating photosynthesis over a diel emersion period and upon changes in light exposure.

## Introduction

Microphytobenthos (MPB) are phototrophic communities of intertidal and neritic benthic ecosystems (MacIntyre et al., 1996; Cahoon, 1999). MPB are responsible for a significant fraction of the total primary productivity of estuaries and coastal ecosystems, mediating nutrient cycling, enhancing benthic–pelagic coupling and efficiently stabilizing the sediment (Underwood and Kromkamp, 1999; Sundbäck et al., 2000; Bellinger et al., 2009). Intertidal MPB communities are largely dominated by diatoms, although other groups of phototrophs may occur, such as cyanobacteria and euglenids. The ecological success of the MPB in intertidal systems has recurrently been linked to cell motility as it allows photosynthetic microbes to search for optimal environmental conditions regarding decisive parameters such as light, temperature or nutrient availability (e.g., Cohn, 2001).

Motile diatoms of intertidal muddy sediments – generally referred to as epipelic diatoms – exhibit migratory rhythms synchronized with diurnal and tidal cycles (Round and Palmer, 1966; Pinckney and Zingmark, 1991). These microalgae accumulate at the sediment surface during diurnal low tides and migrate down into the sediment before tidal inundation or darkness. Upward migration during diurnal low tide periods allows cells to reach the photic zone, causing significant changes in the MPB biomass actually participating in community-level photosynthesis. It has been suggested that downward migration reduces the exposure of cells to predation or physical disturbance and facilitates nutrient and carbon uptake and/or cell division (Admiraal, 1984; Saburova and Polikarpov, 2003). Significant fortnight and seasonal differences in benthic diatom vertical migration were reported and related to the timing of the low tide, previous light history and composition of the diatom populations (Mitbavkar and Anil, 2004; Serôdio et al., 2008). More recently, similar migratory rhythms have been described for MPB inhabiting subtidal sediments, where the diurnal period seems to represent the main trigger for up- and downward migration (Longphuirt et al., 2006; Bourgeois et al., 2010).

In addition to such partially endogenous vertical bulk migration, epipelic diatoms can respond to changes in light levels and migrate downward in the sediment when exposed to high irradiances (Kromkamp et al., 1998; Perkins et al., 2001; Cartaxana et al., 2011). The observation of such photophobic migration has led to the formulation of the “micromigration” theory, a mechanism by which motile MPB would constantly change their position in the sediment light gradient, avoiding photoinhibition and increasing photosynthetic performance (Kromkamp et al., 1998). In accordance, Underwood et al. (2005) reported a sequential species change in sediment surface layers over a diel emersion period. Micromigration could thus represent a rapid, flexible and energetically cheap way of intertidal MPB to optimize productivity (Serôdio et al., 2001). Such mechanism is absent in epipsammic communities of non-motile diatoms dominating more sandy substrata (Jesus et al., 2009).

Photosynthetic organisms may also respond to changes in light exposure through physiological up and down regulation mechanisms, operating on different time scales. Longer-term mechanisms (hours to days), generally designated photoacclimation, involve regulated changes in the levels of photosynthetic pigments, electron transport chain components and carbon metabolism enzymes (MacIntyre et al., 2002; Raven and Geider, 2003). Shorter-term mechanisms (seconds to minutes) involve the regulation of enzymatic activities and include non-photochemical dissipation of excitation energy and damage/repair processes at the level of the photosystem (PS) II reaction centers (Müller et al., 2001; Wu et al., 2011; Cartaxana et al., 2013). In diatoms, the most important physiological photoprotection mechanism is the xanthophyll cycle (XC), involving the de-epoxidation of the pigment diadinoxanthin (Ddx) to diatoxanthin (Dtx) under high light (HL) and the thermal dissipation of harmful excess energy that decrease the excitation rate of PSII reaction centers (Goss and Jakob, 2010). Their exceptionally high capacity for rapid and large induction of the XC cycle under light stress may be a central feature explaining the success of diatoms in variable light environments (Ruban et al., 2004). The relevance of photoprotection through the XC cycle has also been shown for benthic diatoms, both in cell suspensions and in undisturbed MPB natural communities (Serôdio et al., 2005a; Cartaxana et al., 2011).

The microenvironment of densely populated MPB communities is complex and heterogeneous, characterized by steep physical and chemical gradients. Although previous studies have used microsensors to assess MPB migration and primary productivity (e.g., Denis and Desreumaux, 2009; Bourgeois et al., 2010), the simultaneous assessment of the roles of behavioral and photophysiological mechanisms on the regulation of MPB photosynthesis have not been previously determined at relevant spatial scales with minimal disturbance of the photic zone microenvironments. We present such a study using a combination of O_2_ and scalar irradiance microprofiling, variable chlorophyll (Chl) fluorescence and pigment analysis, and address the relevance of photophobic migration and photoprotection via the XC cycle by comparing migratory and non-migratory (treated with a diatom motility inhibitor) benthic biofilms briefly subjected to high irradiance.

## Materials and Methods

### Sediment Sampling and Experimental Set-Up

About 2 cm deep sediment samples were collected during low tide from a muddy intertidal flat in Lisbon, Portugal (38°47′46.7″N, 09°05′32.4″W) on June 2nd 2015, and then transferred with minimal disturbance to custom-made acrylic flow-through chambers. The sediment was composed of more than 99% of particles <63 μm. Microscopic analysis revealed that the MPB community was composed almost exclusively of epipelic diatoms dominated by species of the genera *Navicula*, particularly *N. spartinetensis* and *N.* cf. *phyllepta*. In the laboratory, a stable laminar flow of aerated water collected from the sampling site was maintained above the sediment surface (water layer depth of 1.5 cm; 25°C and a salinity of 25) using a submersible water pump (Rena Flow 400, France). The sediments were kept in the dark overnight. The following day, the water was drained from the flow-through chambers at 8:45 AM and the sediments illuminated homogeneously with an incident downwelling photon irradiance of 150 μmol photons m^-2^ s^-1^ from 9 AM to 1 PM, matching the period of daytime low tide at the site where the sediment was collected. Illumination was done with fiber-optic tungsten halogen lamps (KL-2500, Schott GmbH, Germany) equipped with collimating lenses positioned vertically above the sediment surface. Downwelling photon irradiance of photosynthetically active radiation (PAR, 400–700 nm) was measured with a calibrated photon irradiance meter (ULM-500, Walz GmbH, Germany) equipped with a planar cosine collector (LI-190, LiCor, USA).

At regular time intervals during the diel emersion period, depth profiles of O_2_ concentration and gross photosynthesis were measured with O_2_ microelectrodes (see details below). Rapid light-response curves (RLC) of PSII-derived electron transport rates were also measured at the same time points using variable Chl fluorescence imaging along with sediment sampling of the photic zone (0–0.5 mm) for MPB biomass quantification via pigment analysis. Scalar irradiance depth profile measurements were made using a scalar irradiance microprobe. All the referred methods are described in detail below.

In one of the flow-through chambers, six 1.5-cm diameter plastic rings were carefully placed on the sediment surface around 10:30 AM, defining specific areas for chemical treatment with latrunculin A (Lat A), an inhibitor of diatom motility (Cartaxana et al., 2008). Lat A acts by the dissociation of raphe-associated actin cables and the inhibiting effect is rapidly reversible by washing the diatom cells with fresh medium (Poulsen et al., 1999). Three rings were assigned for control treatment (addition of filtered water from the field site only) and three for Lat A treatment applied as described in detail by Cartaxana and Serôdio (2008), without affecting the biofilm photosynthetic activity or disrupting the photic zone microenvironments. Cartaxana et al. (2008) showed that Lat A treatment had no significant effects on PSII quantum yield and on the light response curve parameters *α* and *ETR*_max_ of benthic diatom cultures. After 30 min at 150 μmol photons m^-2^ s^-1^ (low light, LL) the sediment was subjected to 2000 μmol photons m^-2^ s^-1^ (high light, HL). This change in irradiance was achieved without spectral distortion by adjusting the aperture size on the fiber-optic halogen lamp. Depth profiles of O_2_ concentration and subsequently RLC measurements were then performed on the samples after 30 min of HL exposure, whereafter the 0-0.5 mm sediment surface layer was collected for pigment analysis.

### Pigment Analysis

Sediment samples of the 0-0.5 mm thick surface layer of MPB were collected at regular time intervals over the emersion period using the “crème brûlée” sampler described by Laviale et al. (2015b). The sampler consists of a small stainless steel disk (diameter 1.5 cm) surrounded by a ring of known height, defining a space corresponding to the thickness of sediment to be sampled. The sampler head was immersed in liquid N_2_ prior to careful placement on the surface of the sediment and was then pushed slightly downward until the sampler was filled. The sampler was then removed and its base was scraped to exclude excess of sediment, thus producing a discoid sample with a thickness of 0.5 mm. The filled corer was then briefly reimmersed in liquid N_2_, whereafter the sediment was removed from the sampler and placed in a tube that was stored in liquid N_2_ until transfer to a -80°C freezer. Sediment samples were freeze-dried and photopigments extracted with 90% acetone for 24 h. Chl *a* concentrations were measured as a biomass proxy by spectrophotometry on pigment extracts (Heλios β, Thermo Electron Corp., USA) using the method of Jeffrey and Humphrey (1975).

Pigment analysis of the sediment samples collected in the Lat A experiment were done using High Performance Liquid Chromatography (HPLC; LC10 AVP, Shimadzu, Japan) to determine the concentrations of XC pigments (Ddx and Dtx) in addition to Chl *a*. Pigment extraction and HPLC analysis were performed as described in detail by Cartaxana et al. (2011). Pigments were identified from absorbance spectra and retention times, and pigment concentrations were calculated from peak areas obtained in a photodiode array detector (SPD-M10AVP, Shimadzu). Calibration curves were performed using pure crystalline pigment standards (DHI, Hørsholm, Denmark). The de-epoxidation state (DES) was calculated as:

DES =Dtx/(Ddx+Dtx)

where Dtx and Ddx are the concentrations of diatoxanthin and diadinoxanthin, respectively.

### Microprofiling of O_2_ Concentration and Gross Photosynthesis

Depth profiles of dissolved O_2_ concentrations were measured with fast responding (*t*_90_ <0.5 s) Clark-type microelectrodes (tip diameter ∼25 μm, OX-25, Unisense A/S, Denmark; Revsbech, 1989) in vertical steps of 0.05 mm. The O_2_ microelectrodes were connected to a pA meter (Unisense A/S) and signals were recorded via an USB-interfaced A/D-converter (DCR16, Pyro Science GmbH, Germany) through dedicated PC-controlled data acquisition software (Profix, Pyro Science). The O_2_ microsensors were linearly calibrated at experimental temperature and salinity from measurements in aerated water collected from the sampling site and water made anoxic by addition of sodium sulphite. The O_2_ microsensors were mounted on a motor-driven micromanipulator (MU1, Pyro Science), which was interfaced to a desktop computer and controlled with the Profix software. Surface positioning of the microsensors was done while observing the sediment with a PC-interfaced USB digital microscope (AD7013MZT Dino-Lite, AnMo Electronics Corp., Taiwan). The microsensors were inserted into the sediment at an angle of 45° relatively to the vertical incident light beam. The software automatically accounted for the sensor insertion angle and all depths are given in vertical distances.

Volumetric gross photosynthesis rates (*P*_z_, nmol O_2_ cm^-3^ s^-1^) were measured at 0.1 mm depth intervals using the microelectrode light-dark shift technique (Revsbech and Jørgensen, 1983). In this method, the gross rate of photosynthesis is estimated as the initial O_2_ depletion rate at a specific depth during the first few seconds after light is briefly turned off (see also Glud et al., 1992).

### Light Measurements

Spectral scalar irradiance measurements were done at 0.1 mm depth intervals using a scalar irradiance microprobe connected to a fiber-optic spectrometer (USB2000+, Ocean Optics, USA) that was interfaced to a PC running spectral acquisition software (Spectra Suite, Ocean Optics). The scalar irradiance microprobe consisted of a small diffusing sphere (100 μm diameter) cast on the coated tip of a tapered optical fiber (Lassen et al., 1992; Rickelt et al., 2016). The position and insertion angle of the scalar irradiance probe was controlled as described above for the O_2_ microelectrodes. PAR was obtained by integrating scalar irradiance spectra from 400 to 700 nm. The attenuation coefficient of scalar irradiance, *K*_0_, was calculated from the depth profiles of spectral scalar irradiance as (Kühl, 2005):

$$K_{0}=In(E_{1}/E_{2})/\left(z_{2}-z_{1}\right)$$

where *E*_1_ and *E*_2_ are the scalar irradiance measured at depths z_1_ and z_2_ in the sediment, where z_2_ > z_1_.

### Imaging Variable Chlorophyll Fluorometry

Variable Chl fluorescence was measured using an imaging-PAM fluorometer (IMAG-MINI/R, Walz GmbH, Germany) employing a 640 × 480 pixel resolution CCD camera with a 12 mm objective lens. A LED array with an emission peak at 620 nm provided the measuring beam, the actinic light and the saturation light pulses. Photosynthetic activity was assessed by measurements of rapid light curves (RLC; Ralph and Gademann, 2005) via measurements of the effective PSII quantum yield at 12 intensities of actinic light: 0, 47, 66, 85, 117, 171, 255, 402, 583, 857, 1280, and 1550 μmol photons m^-2^ s^-1^. The duration of each irradiance step was 10 s. Numerical values of the Chl fluorescence parameters were extracted from the digital images using analytical software (Imaging Win, Walz), selecting *a priori* areas of interest (AOI). RLC were constructed by calculating, for each level of actinic light, the relative electron transport rate (r*ETR*) from the delivered actinic photon irradiance (*E*) and the effective quantum yield of PSII (Δ*F*/*F*′_m_) by r*ETR* = *E* × Δ*F*/*F*′_m_. The light response was characterized by fitting the model of Eilers and Peeters (1988) to r*ETR* versus irradiance curves and by estimating the initial slope of the light curve *α* (light utilization coefficient) and *ETR*_max_ (maximum r*ETR*).

### Statistical Analysis

The existence of significant differences in biomass (Chl *a*), volumetric gross photosynthesis in the photic zone, and variable Chl fluorescence RLC parameters (*α* and *ETR*_max_) along the emersion period were tested using one-way analysis of variance (ANOVA). Multiple comparisons among pairs of means were performed using the LSD test. Significant differences after HL exposure in the RLC parameters and pigment content (Chl *a* and DES) between control and Lat A treatments were tested using a *t*-test. Statistical analyses were carried out using IBM SPSS Statistics 22.

## Results

### Diel Changes in MPB Biomass

**FIGURE 1:**
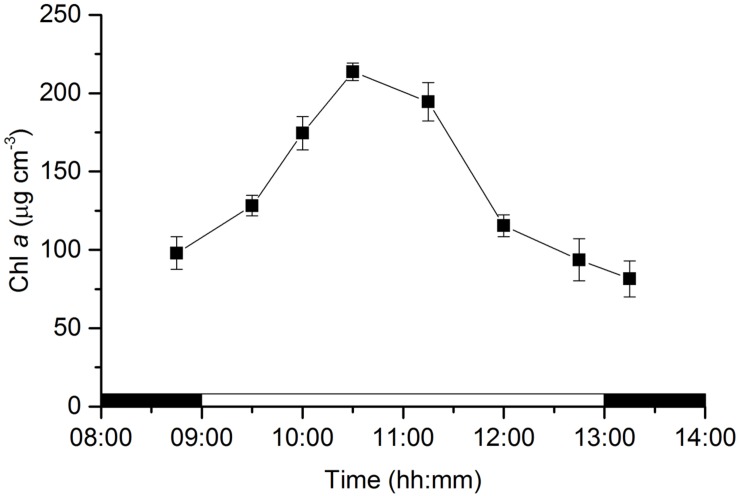
**Changes in microphytobenthos biomass (chlorophyll *a*) in an intertidal sediment photic zone (0–0.5 mm) over a diel emersion period (mean ± standard deviation; *n* = 3).** The illumination period was between 9 AM and 1 PM at a constant photon irradiance of 150 μmol photons m^-2^ s^-1^.

Concentrations of Chl *a* in the photic zone (0–0.5 mm) of the sediment varied significantly along the emersion period (ANOVA, *F*_7,23_ = 74.946, *p* < 0.001). MPB biomass increased during the first half of the emersion period from 97.9 ± 10.4 μg Chl *a* cm^-3^ measured in the dark to 213.6 ± 5.6 μg Chl *a* cm^-3^ at 10:30, 1.5 h after the onset of illumination. Concentrations of Chl *a* started a decreasing trend after 11:15, almost 2 h before the end of the illumination period that coincided with the time of flooding at the sampling site (**Figure 1**).

### Diel Changes in O_2_ Concentration Profiles, Gross Photosynthesis and Variable Chlorophyll Fluorescence Parameters

**FIGURE 2:**
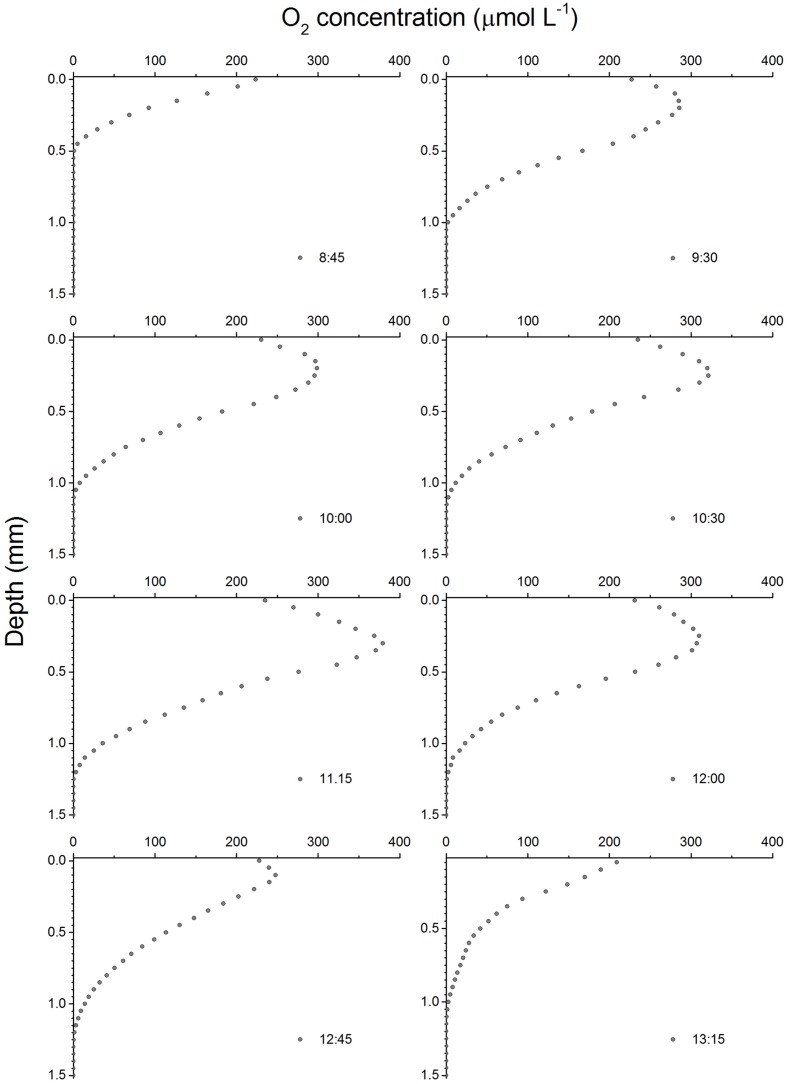
**Depth profiles of O_2_ concentrations in an intertidal sediment over a diel emersion period.** The illumination period was between 9 AM and 1 PM at a constant photon irradiance of 150 μmol photons m^-2^ s^-1^.

Depth profiles of O_2_ concentration changed considerably during the emersion period (**Figure 2**). In the dark, O_2_ concentrations decreased rapidly with depth as result of active O_2_ consumption in the sediment until reaching anoxia at ∼0.5 mm depth. The onset of illumination and the activation of MPB photosynthesis led to a rapid increase in O_2_ concentration reaching a maximum of 285 μM at around 0.1–0.2 mm and decreasing toward deeper sediment layers, becoming undetectable around 1 mm depth. The O_2_ concentration and sediment penetration depth continued to increase during the first half of the emersion period reaching a maximum of 380 μM at 11:15. This increase was accompanied by a change of the maximum O_2_ concentrations to a deeper sediment layer (0.3 mm) and deeper O_2_ penetration depth (1.25 mm). The second half of the emersion period was characterized by a decrease in O_2_ concentrations and a shift back of the O_2_ maximum closer to the sediment surface. Particularly conspicuous was the decrease in O_2_ concentrations at 12:45 (maximum of 248 μM) and the shift of the O_2_ maximum (0.1 mm) toward the end of the illumination period coinciding with the time of flooding at the sampling field site (**Figure 2**).

**FIGURE 3:**
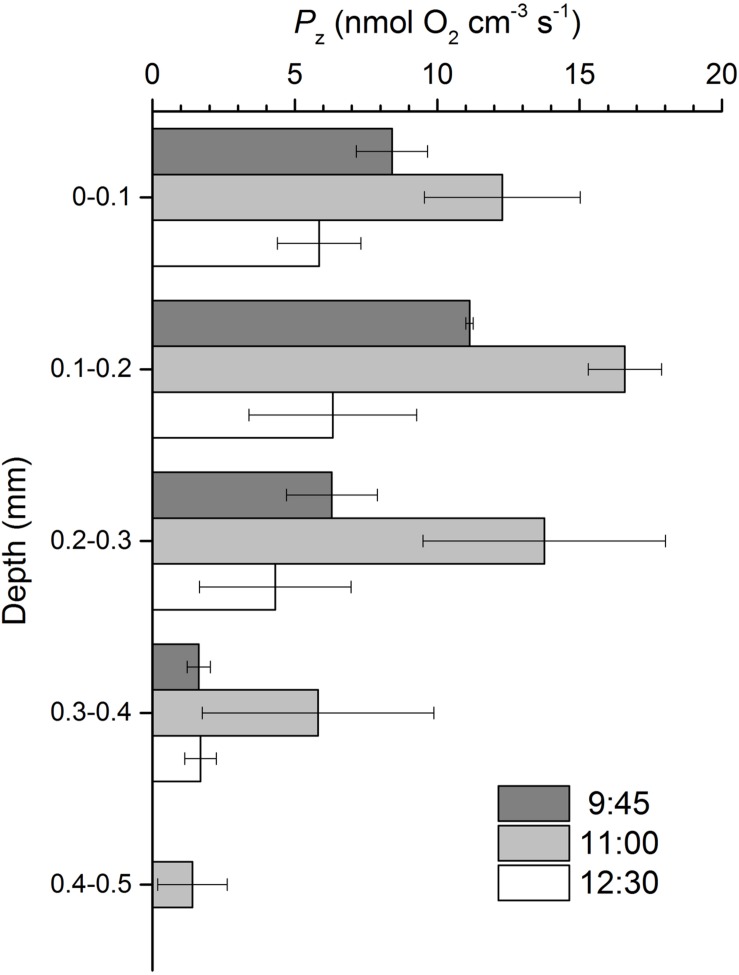
**Gross photosynthesis (*P*_z_) profiles in an intertidal sediment over a diel emersion period (mean ± standard deviation; *n* = 3).** The illumination period was between 9 AM and 1 PM at a constant photon irradiance of 150 μmol photons m^-2^ s^-1^.

Volumetric gross photosynthesis rates showed a similar depth pattern throughout the emersion period, peaking at the 0.1–0.2 mm depth layer and decreasing to undetectable levels between 0.4 and 0.5 mm into the sediment (**Figure 3**). Gross photosynthesis rates reached maximum values of 16.6 ± 1.3 nmol O_2_ cm^-3^ s^-1^ between 0.1 and 0.2 mm at 11:00 (**Figure 3**). Volumetric gross photosynthesis rates averaged for the 0–0.5 mm depth layer varied significantly with time (ANOVA, *F*_2,8_ = 10.933, *p* = 0.010), with higher values observed half-way through the emersion period (11:00) as compared to measurements closer to the onset (9:45) or the end of the illumination period (12:30). Significantly higher rates of O_2_ production in the photic zone of 10.0 ± 2.6 nmol O_2_ cm^-3^ s^-1^ were observed at 11:00 when compared to 9:45 (LSD, *p* = 0.018) and 12:30 (LSD, *p* = 0.004) (**Table 1**). When normalized for Chl *a*, gross photosynthesis was still higher at 11:00, but differences were not statistically significant (ANOVA, *F*_2,8_ = 1.467, *p* = 0.303) (**Table 1**).

**Table 1:** Volumetric and chlorophyll *a*-normalized gross photosynthesis rates averaged for the photic zone (0–0.5 mm) in an intertidal sediment over the diel emersion period (mean ± standard deviation; *n* = 3).

	9:45	11:00	12:30
nmol O_2_ cm^-3^ s^-1^	5.5 ± 0.2^a^	10.0 ± 2.6^b^	3.6 ± 1.4^a^
nmol O_2_ mg^-1^ Chl *a* s^-1^	36.3 ± 1.6	49.6 ± 12.8	36.0 ± 20.5


*The illumination period was between 9 AM and 1 PM at a constant irradiance of 150 μmol photons m^-*2*^ s^-*1*^. Different letters indicate significant differences (LSD) at *p* < 0.05.*

**FIGURE 4:**
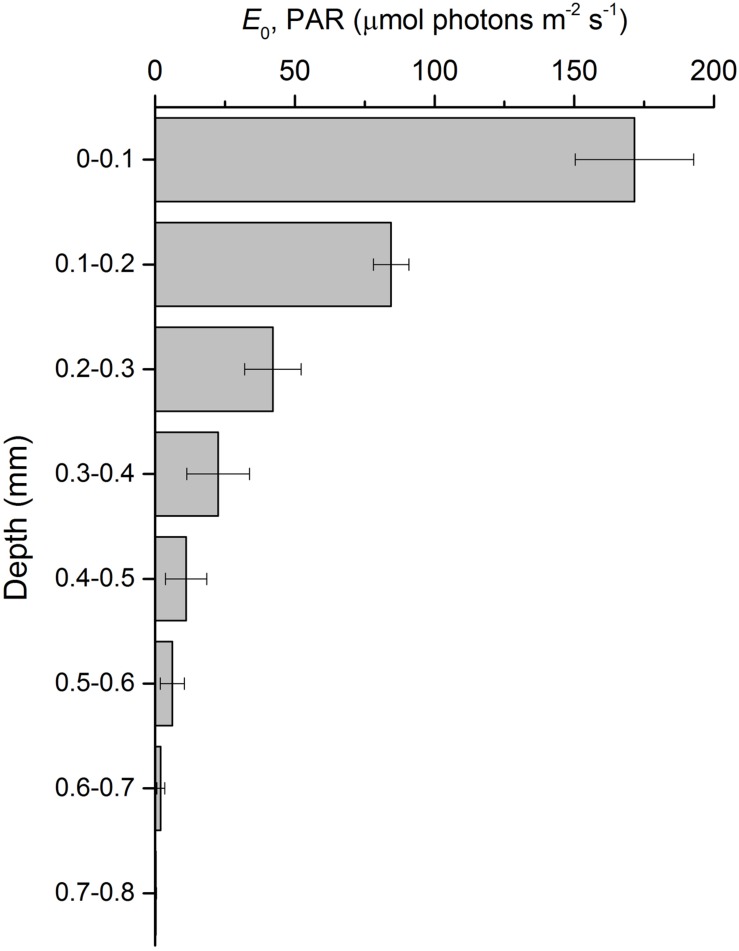
**Scalar irradiance profile of photosynthetically active radiation (*E*_0_, PAR) in an intertidal sediment illuminated at a constant irradiance of 150 μmol photons m^-2^ s^-1^ (mean ± standard deviation; *n* = 3)**.

Depth profiles of scalar irradiance showed strong attenuation of photosynthetic available light with depth (**Figure 4**). Scalar irradiance levels at the sediment surface layer (0–0.1 mm) were higher (*∼*114%) than incident downwelling irradiance, but decreased exponentially to undetectable levels around 0.8 mm. The scalar irradiance attenuation coefficient of PAR, *K*_0_, was 8.2 mm^-1^.

**FIGURE 5:**
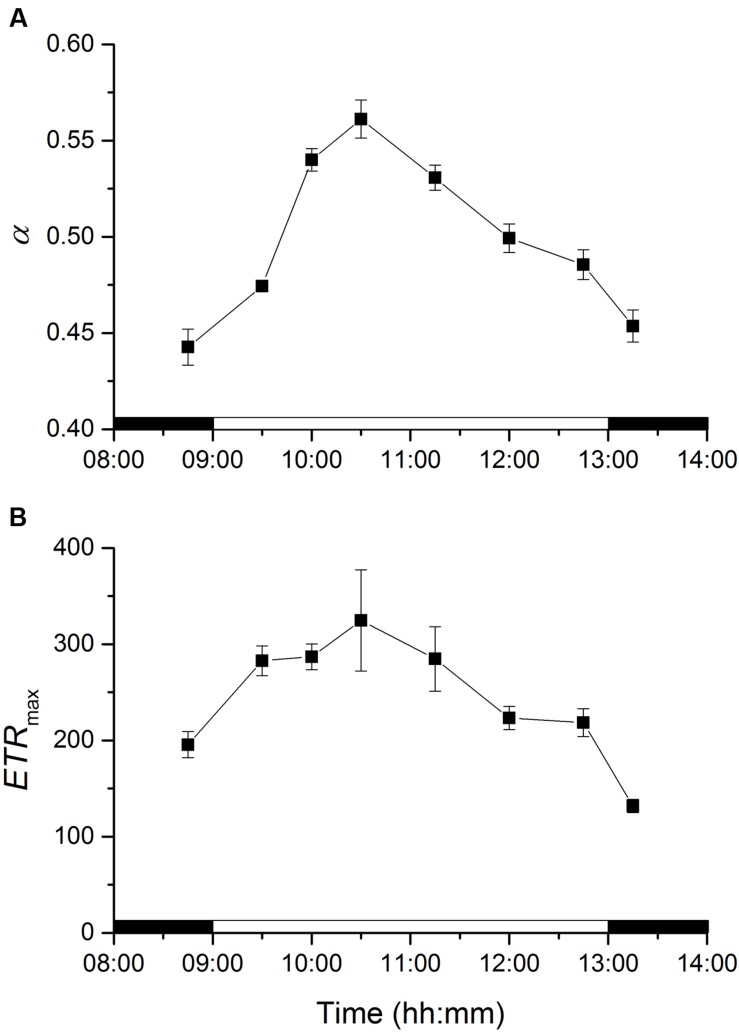
**Changes in rapid light curve parameters *α* (light utilization coefficient, **A)** and *ETR*_max_ (relative maximum electron transport rate, **B)** of an intertidal sediment over a diel emersion period (mean ± standard deviation; *n* = 3).** The illumination period was between 9 AM and 1 PM at a constant photon irradiance of 150 μmol photons m^-2^ s^-1^.

Photosynthetic efficiencies at limiting irradiances, *α*, varied significantly along the emersion period (ANOVA, *F*_7,23_ = 95.872, *p* < 0.001). *α* increased with the transition from dark to light and continued to increase significantly for 90 min after onset of illumination (until 10:30), and then decreased for the rest of the emersion period and after the transition to darkness (**Figure 5A**). Photosynthetic capacities at saturating irradiances (*ETR*_max_) varied significantly along the emersion period (ANOVA, *F*_7,23_ = 19.229, *p* < 0.001). Maximum r*ETR* increased with the transition from dark to light and remained relatively constant from 9:30 to 11:15, and then decreased for the rest of the emersion period and after the transition to darkness (**Figure 5B**).

### Effects of High Light Exposure

**FIGURE 6:**
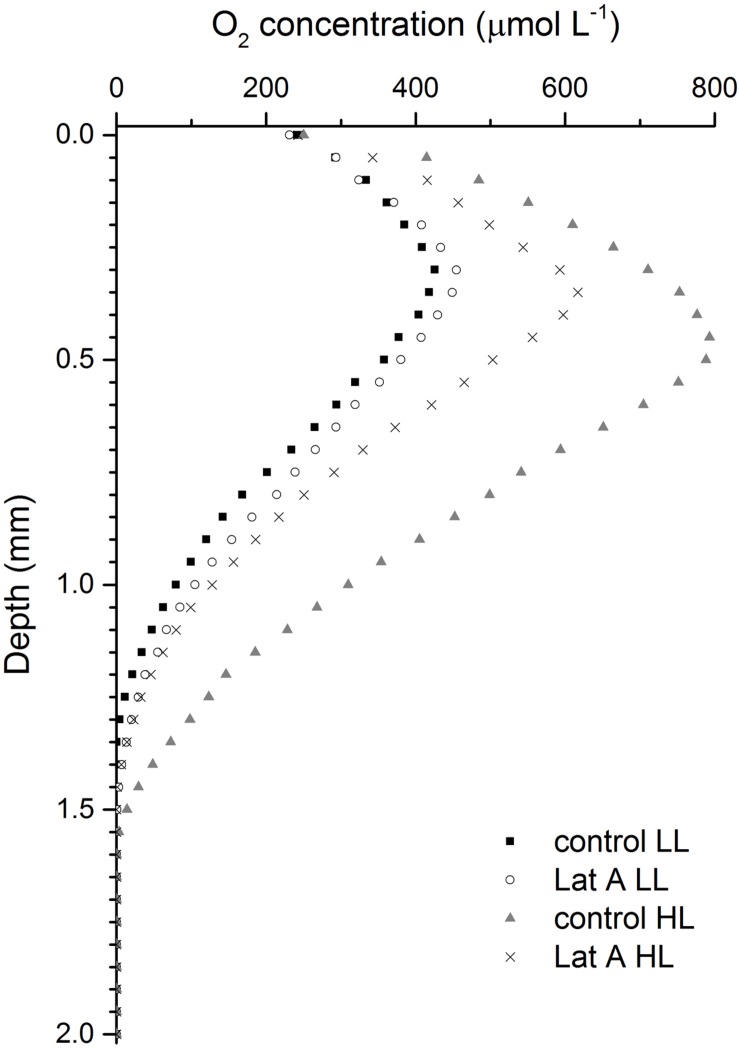
**Depth profiles of O_2_ concentrations for untreated (control) and latrunculin A (Lat A) treated intertidal sediments under low light (LL, 150 μmol photons m^-2^ s^-1^) and after 30 min exposure to high light (HL, 2000 μmol photons m^-2^ s^-1^)**.

Depth profiles of O_2_ concentration were considerably different depending on light (LL vs. HL) and chemical treatments (control vs. Lat A) (**Figure 6**). Under LL, O_2_ profiles were similar in both control and Lat A treatments reaching similar maximum concentrations (∼430 μmol L^-1^) at an identical depth (0.3 mm). During exposure to HL, profiles changed significantly reaching higher O_2_ concentrations under both control and Lat A treatments. However, the increase in O_2_ concentrations was more pronounced in control treatment, reaching higher maximum values (∼800 μmol L^-1^) at a deeper layer (0.45 mm) as compared to Lat A treated samples (∼600 μmol L^-1^ at 0.35 mm depth) (**Figure 6**).

**FIGURE 7:**
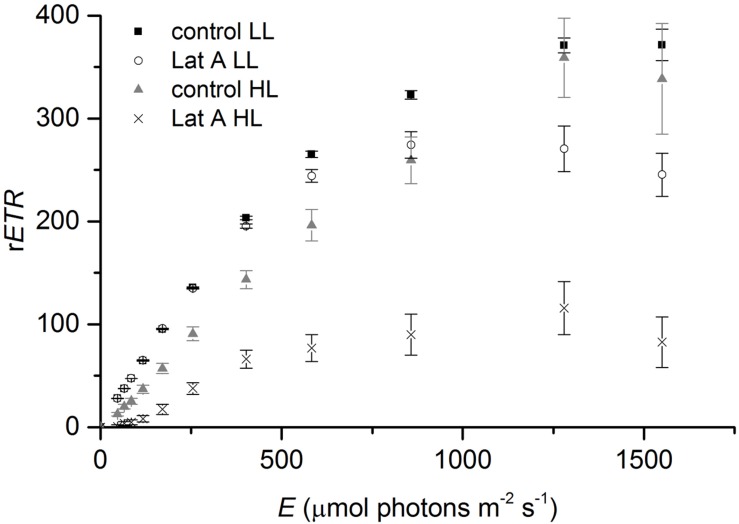
**Rapid light-response curves of relative electron transport rate (r*ETR*) vs. irradiance for untreated (control) and Lat A treated intertidal sediments under low light (LL, 150 μmol photons m^-2^ s^-1^) and after 30 min of exposure to high irradiance (HL, 2000 μmol photons m^-2^ s^-1^)**.

Light (LL vs. HL) and chemical treatments (control vs. Lat A) also significantly affected the shape of the rapid-light response curves (**Figure 7**). For *α*, values were similar under LL (0.584 ± 0.010 and 0.574 ± 0.003 for control and Lat A, respectively) and decreased after the HL treatment. Light utilization coefficient was significantly lower in Lat A HL treatment than in control HL (*t*-test, *p* < 0.001; **Table 2**). For *ETR*_max_, a Lat A treatment effect was observed under LL, with lower *ETR*_max_ values (277 ± 16) when compared to the control (373 ± 13). Maximum r*ETR* was significantly lower in Lat A HL treatment than in control HL (*t*-test, *p* = 0.003; **Table 2**). Average concentration of Chl *a* in the photic zone (0–0.5 mm) was higher in HL Lat A than in control HL, but the difference was not statistically significant (*t*-test, *p* = 0.265; **Table 2**). DES [=Dtx/(Ddx+Dtx)] was significantly higher in HL Lat A than in control HL (*t*-test, *p* = 0.022; **Table 2**), whereas no significant differences were found for the pigment ratio (Ddx+Dtx)/Chl *a* (*t*-test, *p* = 0.311; **Table 2**).

**Table 2:** Pigment composition and photosynthesis vs. irradiance curve parameters (mean ± standard deviation; *n* = 3) of control and latrunculin A (Lat A) treated intertidal sediments after 30 min of exposure to high light (2000 μmol photons m^-2^ s^-1^).

	Control		Lat A
Chl *a* (mg cm^-3^)	0.19 ± 0.07		0.25 ± 0.04
Dtx/(Dtx+Ddx)	0.39 ± 0.07	^∗^	0.59 ± 0.07
(Dtx+Ddx)/Chl *a*	0.127 ± 0.007		0.133 ± 0.006
*α*	0.367 ± 0.023	^∗∗∗^	0.157 ± 0.024
*ETR*_max_	370 ± 67	^∗∗^	99 ± 24


*Pigments were quantified for the photic zone (0–0.5 mm). Chl *a*, chlorophyll *a*; Dtx, diatoxanthin; Ddx, diadinoxanthin; *α*, light utilization coefficient; *ETR*_max_, relative maximum electron transport rate. ^∗^, ^∗∗^, and ^∗∗∗^ indicate significant differences (*t*-test) at *p* < 0.05, 0.01, and 0.001, respectively.*

## Discussion

The MPB biomass in the photic zone (0–0.5 mm) of the studied intertidal sediment showed strong diel variability with sediment Chl concentrations showing a more than 2-fold increase and reaching maximal values of *∼*0.2 mg Chl *a* cm^-3^. These changes can be attributed to the migration of diatom cells from deeper layers to the uppermost 0.5 mm of the sediment, allowing cells to absorb light to drive photosynthesis. A very strong attenuation of photosynthetic available light with depth was observed, and irradiance levels below 0.5 mm were <5% of incident downwelling irradiance. Migratory rhythms of the MPB populations of intertidal mudflats synchronized with diurnal and tidal cycles, commonly identified by the appearance of a golden brown color at the sediment surface, have been frequently described in the literature (e.g., Round and Palmer, 1966; Pinckney and Zingmark, 1991) and were reviewed by Consalvey et al. (2004). More recently, Coelho et al. (2011) presented evidence of a two-phase process in the formation of such densely populated surface diatom biofilms: the first phase being endogenously controlled and beginning before the daytime emersion period, and the second phase driven by environmental factors, especially favored by LL exposure. We observed that the MPB biomass in the photic zone before the onset of illumination (8:45) was higher than late in the illumination period (12:45) and after the transition to darkness (13:15), supporting the hypothesis that an endogenously driven upward MPB cell migration to the sediment surface began before the start of the emersion period, when the sediment was still water covered and in the dark, probably in anticipation of a favorable period for photosynthesis (Coelho et al., 2011).

Particularly interesting was the observation that maximal Chl *a* values in the surface biofilm were reached early in the morning 1.5 h after the onset of illumination, whereafter concentrations started to decrease almost 2 h before the end of the illumination period. A possible reason for intertidal MPB communities to avoid midday summer exposure is the extremely high temperatures observed in the sediment surface. In the Tagus estuary, if emersion coincides with summer midday, the exposed dark-colored mudflat sediment surface can reach temperatures of > 35°C (Serôdio and Catarino, 1999). Downward migration would thus avoid exposure to simultaneous high irradiance and temperature stress found in the uppermost sediment layer. Laviale et al. (2015a) observed that higher temperature significantly increased susceptibility to photoinhibition in diatom-dominated MPB communities. Considering that our study was done under controlled and constant low irradiance (150 μmol photons m^-2^ s^-1^) and optimum temperature conditions (25°C), such migratory response would require endogenous control on top of the circadian and tidal rhythms. However, other factors could be driving downward migration early in the diurnal emersion period. For example, Saburova and Polikarpov (2003) observed that epipelic diatom cells in different phases of mitosis were found almost exclusively in the aphotic zone of the sediment and argued that deeper sediment layers provide more favorable nutrient conditions for cell growth and division.

This study reports a diel pattern in intertidal sediment O_2_ concentrations and MPB photosynthesis rates in the photic zone under constant LL over an emersion period, with an increase during the first hours of illumination and a decrease during the second half of the exposure period. Using O_2_ microelectrodes, Brotas et al. (2003) reported comparable changes in MPB photosynthetic rates *in situ*, with an increase from the beginning of the illumination period until it reached a plateau for 2 h, followed by a pronounced decrease before the end of the exposure period. The results suggest that MPB community-level photosynthesis is mainly controlled by changes in the productive biomass of the photic zone due to diatom migration. Interestingly, gross photosynthesis reached highest rates below the surface (0.1–0.2 mm), although light was strongly attenuated with depth. This can be the result of a higher accumulation of MPB cells at this subsurface sediment layer or result from a limitation of the microelectrode light-dark shift method in non-submerged biofilms due to rapid removal of gaseous species at the biofilm interface that leads to underestimation of photosynthetic rates at the uppermost sediment layer (Li et al., 2015).

Variable Chl fluorescence-derived measures of photosynthesis vs. irradiance curves are commonly used as a way of characterizing the photoacclimation status of MPB (e.g., Kromkamp et al., 1998; Serôdio et al., 2005b). However, interpretation of variable Chl fluorescence measurements on intact MPB is complex as fundamental assumptions are not verified in optically dense microalgal biofilms. Due to the vertical attenuation of downwelling measuring, actinic, and saturating light, and of upwelling fluorescence, the fluorescence levels measured at the sediment surface include the contributions of emissions at different depths, which are exposed to irradiance levels different from those measured at the surface (Serôdio, 2004). Consequently, the quantum yield calculated from depth-integrated measurements may differ from the cells’ intrinsic Δ*F*/*F*′_m_. Furthermore, Δ*F*/*F*′_m_ can be affected by changes in the composition of the biofilms resulting from natural migratory rhythms or vertical movements induced during the measurements under increasing irradiance (Perkins et al., 2002). In this study, we used fluorescence rapid-light curves (RLC) with light steps of 10 s that can be completed within 2 min, minimizing the confounding effects of vertical cell migration (Serôdio et al., 2005b). However, due to strong light attenuation in the sediment (*K*_0_ = 8.2 mm^-1^), estimation of Δ*F*/*F*′_m_ could still be affected by only minor changes of the cells within the vertical profile during the RLC. Diatom cells coming from deeper sediment layers that were previously exposed to lower light and therefore have higher photosynthetic yields are continuously reaching the surface. At the same time, cells at the surface are shaded by upward migrating cells whereas others actively migrate down, being suddenly exposed to significantly less light. This is perceptible on the light-saturating part of the RLC when comparing Lat A and control treatments kept under LL (150 μmol photons m^-2^ s^-1^) because (i) vertical migration could be amplified by the higher irradiances (*E*) of the final light steps of the RLC; (ii) *ETR* is calculated by multiplication of *E* × Δ*F*/*F*′_m_, so minor changes in Δ*F*/*F*′_m_ induced by migration would be amplified at higher *E* values.

A diel pattern in the RLC photosynthetic parameters *α* (photosynthetic efficiency at limiting irradiance) and *ETR*_max_ (photosynthetic capacity at saturating irradiance) was observed in the present study. By comparing measurements in undisturbed intact samples with cell suspensions and sediment slurries, Serôdio et al. (2005b) concluded that diel patterns in RLC measured in MPB throughout a dark-light cycle were relatively independent of changes in the vertical distribution of microalgae and caused mainly by changes at the physiological level. Using high-resolution single-cell fluorescence imaging, Underwood et al. (2005) reported that individual MPB taxa present at the surface layers of intertidal biofilms showed down-regulation of photosynthetic efficiency over a diel emersion period, which mirrored the overall biofilm functioning. The authors speculated that higher efficiencies during the earlier part of the photoperiod could represent a strategy by MBP to maximize photosynthesis as soon as the diatoms arrive at the surface.

Variations in light-limited photosynthesis are known to be associated to changes in the light-harvesting complexes and/or the activity of the photosynthetic light reactions and PSII (Behrenfeld et al., 2004). Leblanc et al. (1999) investigated photoregulation of the expression of a gene encoding a fucoxanthin Chl *a/c* binding protein (*fcp*), one of the major components of the PS II-associated light harvesting complex in diatoms, and reported a 5- to 6- fold increase in mRNA levels of dark-adapted *Thalassiosira weissflogii* cells when exposed to illumination. Meyer et al. (2003) reported a diel pattern in *fcp* mRNA levels in a MPB natural community dominated by pennate diatoms: levels increased with sunrise and peaked between 10:00 and 12:00 AM, and decreased through the rest of the illumination period. In diatoms, it has been shown that the chlororespiratory electron flow in darkness is sufficient to establish a proton gradient across the thylakoid membrane, thereby leading to an activated XC and the accumulation of Dtx increasing non-photochemical quenching (NPQ; Jakob et al., 1999). Hence, the observed increase in *α* during the transition from darkness to LL can also be partially attributed to the dissipation of NPQ formed in the dark. Changes in light-saturated photosynthesis are usually attributed to processes “down-stream” of PSII, namely the concentration and/or activity of the Calvin cycle enzyme ribulose-1,5-bisphosphate carboxylase/oxygenase (RUBISCO) and photosynthetic electron transport components (Behrenfeld et al., 2004). A consistent pattern of diel transcriptional regulation of RUBISCO has been shown for natural phytoplankton populations, with transcript levels of the large subunit (*rbc*L) increasing early in the light period and diminishing toward the end of the light phase and into the dark period (Pichard et al., 1996). Wawrik et al. (2002) observed a diurnal regulation on *rbc*L mRNA expression in the diatom *Phaeodactylum tricornutum*, increasing before the light period and peaking during the early morning followed by a significant decrease in the afternoon. Hence, diel changes at the physiological level may help explain the observed patterns in MPB photosynthetic parameters.

Under HL exposure (2000 μmol photons m^-2^ s^-1^), we observed lower *α*, *ETR*_max_ and sediment O_2_ concentrations when cell migration was inhibited with the diatom motility inhibitor Lat A, showing that migration is also used by MPB to maximize photosynthesis by reducing exposure to potentially photoinhibitory light levels. Although concentrations of Chl *a* in the photic zone were higher in non-motile biofilms after HL treatment, differences were not statistically significant from control biofilms. This indicates that HL mainly causes reallocation of cells within the photic zone to more optimal light conditions and not necessarily a massive migration of MPB to deeper aphotic layers (Cartaxana et al., 2011). Using the same motility inhibitor, Perkins et al. (2010) identified vertical movement as the primary response of epipelic diatoms of muddy sediments to accumulated light dose, in accordance with the “micromigration” theory (Kromkamp et al., 1998). Contrary, Serôdio et al. (2012) estimated that epipelic diatom biofilms treated with Lat A showed only a *∼*10% higher photoinhibition than migratory biofilms and suggested that physiological processes were more important for photoprotection. In our study, the significant higher DES in the photic layer of Lat A-treated biofilms under high irradiances indicates that the XC is more relevant for photoprotection when epipelic diatom cells are unable to migrate. The fact that the (Dtx+Ddx)/Chl *a* ratio was constant between treatments confirms that Dtx is formed from the de-epoxidation of Ddx through the XC and not by *de novo* synthesis. A relationship between growth-form and photoprotection mechanisms in intertidal benthic diatoms was proposed by Cartaxana et al. (2011) suggesting that epipelic motile diatoms of muddy sediments can photoregulate via both physiological and behavioral photoprotection, while non-motile epipsammic diatoms of sandy sediments exclusively rely on physiological mechanisms. Analyzing the photophysiological traits of 15 marine benthic diatom species, Barnett et al. (2015) showed an increasing trend of NPQ and DES from motile epipelic to non-motile epipsammic species.

## Conclusion

We report a diel pattern of photosynthesis over an emersion period for an intertidal diatom-dominated MPB community that was mainly determined by cell migration and changes in the productive biomass of the photic zone. This behavioral trait is also used by MPB to maximize photosynthesis under exposure to potentially photoinhibitory light levels.

## Author Contributions

PC, SC, and MK designed research and outlined experiments. PC, SC, and CG conducted the experiments. All authors analyzed and interpreted the data. PC wrote the manuscript. All authors read, critically revised and approved the final version of the manuscript.

## Conflict of Interest Statement

The authors declare that the research was conducted in the absence of any commercial or financial relationships that could be construed as a potential conflict of interest.
